# An Innovative Time-Cost-Quality Tradeoff Modeling of Building Construction Project Based on Resource Allocation

**DOI:** 10.1155/2014/673248

**Published:** 2014-01-30

**Authors:** Wenfa Hu, Xinhua He

**Affiliations:** ^1^School of Economics and Management, Tongji University, Shanghai 200092, China; ^2^School of Economics Management, Shanghai Maritime University, Shanghai 201306, China

## Abstract

The time, quality, and cost are three important but contradictive objectives in a building construction project. It is a tough challenge for project managers to optimize them since they are different parameters. This paper presents a time-cost-quality optimization model that enables managers to optimize multiobjectives. The model is from the project breakdown structure method where task resources in a construction project are divided into a series of activities and further into construction labors, materials, equipment, and administration. The resources utilized in a construction activity would eventually determine its construction time, cost, and quality, and a complex time-cost-quality trade-off model is finally generated based on correlations between construction activities. A genetic algorithm tool is applied in the model to solve the comprehensive nonlinear time-cost-quality problems. Building of a three-storey house is an example to illustrate the implementation of the model, demonstrate its advantages in optimizing trade-off of construction time, cost, and quality, and help make a winning decision in construction practices. The computational time-cost-quality curves in visual graphics from the case study prove traditional cost-time assumptions reasonable and also prove this time-cost-quality trade-off model sophisticated.

## 1. Introduction

The time, quality, and cost are usually three contradictive objectives which are often traded off in project practices by managers randomly if they lack efficient tools. The time, quality, and cost are interdependent parameters in a building project.

When the construction time is shortened, the project cost should be added. It is a tough challenge to balance those objectives in practice. The cost is usually the most important determinant of selecting a contractor in current construction industry. A contractor is undergoing fewer profit margins now than ever when current construction industry is more competitive. He might lose all profit or even go bankrupt if he fails to implement one or two projects properly in right quality, time, and cost. In order to reduce cost, some contractors risk using inferior construction materials and incapable labor which frequently results in poor quality and thus compromise safety standards. Local government offices have to monitor and regulate construction quality to secure the minimum standards. Otherwise any contractor might be punished or ousted by governments if he fails to obey regulations of construction quality and safety. Besides, the time is such a top visible parameter that contractors would deliver the construction project within their promised schedule based on agreements and contracts. Obviously the time is sensitive in contracting projects and controlling cost. A construction manager should deliberately balance the cost, time, and quality, as well as construction resources, in the early planning phase.

Since the cost and time are two of the most important objectives which are easily quantified in a construction project, time-cost tradeoff problem has been researched for a long time [1]. Basic common assumptions for cost-time tradeoff are deterministic durations and linear time-cost functions where discrete construction resources such as labor and machine are crashed up to a continuous extent. In order to reach global optimum in a large-scale time-cost tradeoff model, numerous trials are usually needed. There are more than 23 optimal techniques thought to be the most effective in achieving time-cost tradeoff problems [2]. The evolutionary algorithms are more efficient to avoid local optimization. A practical method to solve construction time-cost tradeoff problems is the genetic algorithm (GA) [3, 4]. Recently the ant colony optimization (ACO) algorithm and the particle swarm optimization algorithm are applied to obtain a global optimization solution [5, 6]. Geem [7] employed the harmony search algorithm to perform time-cost biobjective tradeoff where a network of up to 18 nodes was tested well. These new paradigm algorithms were able to obtain optimal solutions for cost-time tradeoff models within only moderate computational effort.

Quality is an important parameter correlating highly with time and cost parameters. But it is not a quantitative parameter in nature, practical time-cost-quality tradeoff models are seldom developed from previous research works of the literature. Babu and Suresh [8] proposed a framework to study the tradeoff among time, cost, and quality using three interrelated linear programming models. Then Khang and Myint [9] applied the linear programming models in an actual cement factory construction project, which was depicted by a 52-activity CPM incorporated with their time, cost, and quality individually, and quality parameter in every activity varied from 0.85 to 1. Tareghian and Taheri [10] assumed the duration and quality of project activities to be discrete and developed a three interrelated integer programming model but simplified the optimization algorithm with solving one of the given entities by assigning desired bounds on the other two. El-Rayes and Kandil [11] presented a multiobjective model to search for an optimal resource utilization plan that minimizes construction cost and time while maximizing its quality, applied genetic algorithm to provide the capability of quantifying and considering quality, and visualized optimal tradeoffs among construction time, cost, and quality by an application example.

Although the objectives of cost and time might be mentioned frequently by natural numbers, the objective of quality is seldom described in quantities, which worsens numerical tradeoff among project time, cost, and quality. This paper will present a new solution for solving time-cost-quality tradeoff problem based on project breakdown structure method and task resource allocation.

## 2. Problem Definition

Project time-cost-quality tradeoff problem (PTCQTP) can be defined as follows: a project is represented by an activity-on-node network with *n* activities that is an acyclic digraph *G* = (*A*), where *A* = {0,…, *n* + 1} is the set of nodes (construction activities). In the network both node (0) and node (*n* + 1) are dummy activities. *P* is the set of all paths in the activity-on-node network, starting from activity (0) and ending at activity (*n* + 1) and *P*_*l*_ is the set of activities contained in path *l* ∈ *P*.

Each activity *i* ∈ *A* is associated with its time *T*_*i*_, cost *C*_*i*_, and quality *Q*_*i*_. The earliest/latest starting times (EST_*i*_^*s*^/LST_*i*_^*s*^) for each activity *i* are easily calculated using the forward-backward passes. Each activity *i* can be decomposed into four resources of *L*_*i*_ (construction labor), *M*_*i*_ (construction material and machine), *E*_*i*_ (construction equipment), and *A*_*i*_ (construction administration). Construction labor *L*_*i*_ is associated with labor productivity LP_*i*_, labor cost LC_*i*_, labor amount LA_*i*_, and labor quality LQ_*i*_. Construction material *M*_*i*_ is associated with material and machine cost MC_*i*_ and material quality MQ_*i*_. Construction equipment *E*_*i*_ is associated with equipment productivity *EP*⁡_*i*_, equipment cost EC_*i*_, equipment amount EA_*i*_, and equipment quality EQ_*i*_. Construction administration *A*_*i*_ is associated with administration cost AC⁡_*i*_ and administration quality AQ_*i*_.

In order to guarantee public safety and interest, local governments would supervise and secure all construction projects to be above a minimum quality level (*Q* ^min⁡^) [12]. If any part of a construction project fails to conform with the minimum construction quality standards, the project could not be delivered properly, and the unqualified parts (LQ_*i*_, MQ_*i*_, EQ_*i*_) should be replaced or reworked until this quality conforms to the minimum requirements such as the minimum labor quality (LQ_*i*_^min⁡^), the minimum material quality (MQ_*i*_^min⁡^), the minimum equipment quality (EQ_*i*_^min⁡^), and the minimum administration quality (AQ_*i*_^min⁡^). The reworks or replacement of construction parts obviously increase cost and delay schedule if the parts of inferior quality are detected by supervisors according to regulations and codes [12]. Research on rework or replacement is so complicated that it would mislead this paper into game method rather than optimization analysis, so this model assumes that any part of a construction part could not be below its minimum standard.

Since the project delivery time defined clearly in construction agreements is a crucial factor for project owner and contractors, contractors should complete and deliver the project to the owner in time. Otherwise the contractors will pay a certain penalty because of delay delivery [13]. This kind of contract conditions will encourage the contractor to set up a project time plan in advance. Contractors are naturally interested in controlling cost actively and minimize the project total cost (*C*).

As discussed previously in aspects of local government's regulations in securing construction quality, project owner's stimulus to shorten construction time, and contractor's intrinsic motivation to reduce construction cost, PTCQTP can be formally stated as follows: given a network with a lot of nodes, that is, activities by their sequences, durations, costs, and qualities, a general status is determined by each activity according to at least one of the following objectives: minimize the project duration, maximize the requirements of construction quality codes and standards, and minimize budget.

## 3. Decision Variables and Assumptions

**Figure 1:**
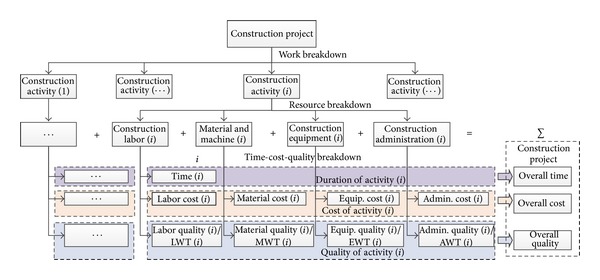
Breakdown and formation structure of time-cost-quality in a construction project essential relationships among the time, cost, and quality parameters in construction activities are assumed and discussed as follows.

The project time-cost-quality performance is essentially formed from each activity's time, cost, and quality, respectively. The work breakdown structure (WBS) is a method to decompose a project into a number of construction activities and further into construction resources such as labors, materials, equipment, and administration, whose utilization determines each activity's time, cost, and quality parameters and the project's overall time/cost/quality performance is finally formed, as shown in Figure 1.

### 3.1. Relationship between Labor Productivity and Labor Quality

Assign a construction team to excavate earth, to erect formwork, to mix concrete, or to do other jobs; their working quality will decline if they intend to increase productivity [14], and an approximate linear relationship between labor productivity and labor quality is observed here. A more complex relationship function between labor productivity and labor quality is also considered in later case study:

(1) $$\begin{array}{c} {\text{LPRD}}_{\left(i\right)}={\text{LPRD}}_{i}^{\max}-{\text{LQK}}_{i}\times \left({\text{LQ}}_{\left(i\right)}-{\text{LQ}}_{i}^{\min}\right), \end{array}$$

where LQ_(*i*)_ = actual quality level of construction labor (*i*) working in activity (*i*), LQ_(*i*)_ ∈ (LQ_*i*_^min⁡^, LQ_*i*_^max⁡^); LQ_*i*_^max⁡^ = maximum quality level of construction labor (*i*) working in activity (*i*); LQK_*i*_ = (LPRD_*i*_^max⁡^ − LPRD_*i*_^min⁡^)/(LQ_*i*_^max⁡^ − LQ_*i*_^min⁡^); LPRD_*i*_^min⁡^ = minimum productivity level of construction labor (*i*) working in activity (*i*); LPRD_*i*_^max⁡^ = maximum productivity level of construction labor (*i*) working in activity (*i*); LPRD_(*i*)_ = actual productivity level of construction labor (*i*) working in activity (*i*), LPRD_(*i*)_ ∈ (LPRD_*i*_^min⁡^, LPRD_*i*_^max⁡^).

### 3.2. Relationship between Material Quality and Material Cost

The time of a construction activity is mainly determined by its job quantities and productivities rather than its material or machine quality, and the material can hardly interfere with the activity's time, either [15]. Thereafter an approximate linear relationship between material quality and material cost is determined.

The relationship between quality and quality cost for a manufacturing company

(2) $$\begin{array}{c} {\text{MC}}_{\left(i\right)}={\text{MC}}_{i}^{\min}+{\text{MQK}}_{i}\times \left({\text{MQ}}_{\left(i\right)}-{\text{MQ}}_{i}^{\min}\right), \end{array}$$

where MQ_(*i*)_ = actual quality level of construction material in activity (*i*), MQ_(*i*)_ ∈ (MQ_*i*_^min⁡^, MQ_*i*_^max⁡^); MQ_*i*_^min⁡^ = minimum quality level of construction material in activity (*i*); MQ_*i*_^max⁡^ = maximum quality level of construction material in activity (*i*); MQK_*i*_ = (MC_*i*_^max⁡^ − MC_*i*_^min⁡^)/(MQ_*i*_^max⁡^ − MQ_*i*_^min⁡^); MC_*i*_^min⁡^ = minimum cost of construction material in activity (*i*); MC_*i*_^max⁡^ = maximum cost of construction material in activity (*i*); MC_(*i*)_ = actual cost of construction material in activity (*i*), MC_(*i*)_ ∈ (MC_*i*_^min⁡^, MC_*i*_^max⁡^).

### 3.3. Relationship among Equipment Productivity, Quality, and Cost

Construction equipment is a crucial factor of construction techniques to increase construction quality, to reduce cost, and to shorten time. In order to calculate construction time variation impacted by equipment, a modified factor to labor productivity caused by equipment (*i*) is introduced [14]:

(3) $$\begin{array}{c} {\text{PRD}}_{\left(i\right)}={\text{LPRD}}_{\left(i\right)}\times {\text{DEK}}_{\left(i\right)}, \end{array}$$

where PRD_(*i*)_ is the actual productivity in activity (*i*); DEK_(*i*)_ is a modified factor to labor (*i*) productivity by changes of construction equipment parameters; LPRD_(*i*)_ is labor productivity in activity (*i*).

A better equipment quality performance will improve construction productivity [15], so the modified factor DEK_(*i*)_ could be derived from the equipment quality EQ_(*i*)_:

(4) $$\begin{array}{c} {\text{DEK}}_{\left(i\right)}={\text{DEK}}_{i}^{\min}+{\text{DQK}}_{i}\times \left({\text{EQ}}_{\left(i\right)}-{\text{EQ}}_{i}^{\min}\right), \end{array}$$

where DQK_*i*_ = (DEK_*i*_^max⁡^ − DEK_*i*_^min⁡^)/(EQ_*i*_^max⁡^ − EQ_*i*_^min⁡^).

Construction equipment quality and equipment cost [15] is also assumed as an approximate linear function just like construction material:

(5) $$\begin{array}{c} {\text{EC}}_{\left(i\right)}=\left[{\text{EC}}_{i}^{\min}+{\text{EQK}}_{i}\times \left({\text{EQ}}_{\left(i\right)}-{\text{EQ}}_{i}^{\min}\right)\right], \end{array}$$

where EQ_(*i*)_= actual quality level of construction equipment (*i*) in activity (*i*), EQ_(*i*)_ ∈ (EQ_*i*_^min⁡^, EQ_*i*_^max⁡^); EQ_*i*_^min⁡^ = minimum quality level of construction equipment (*i*) in activity (*i*); EQ_*i*_^max⁡^ = maximum quality level of construction equipment (*i*) in activity (*i*); EQK_*i*_ = (EC_*i*_^max⁡^ − EC_*i*_^min⁡^)/(EQ_*i*_^max⁡^ − EQ_*i*_^min⁡^); EC_*i*_^min⁡^ = minimum cost of construction equipment (*i*) in activity (*i*); EC_*i*_^max⁡^ = maximum cost of construction equipment (*i*) in activity (*i*); EC_(*i*)_ = actual cost of construction equipment in activity (*i*), EC_(*i*)_ ∈ (EC_*i*_^min⁡^, EC_*i*_^max⁡^).

Work overtime usually decreases construction productivity and increases hourly cost rate [16]. Then construction equipment cost EC_(*i*)_ will be modified by factor *α*_*i*_:

(6) EC(i)=[ECimin⁡+EQKi×(EQ(i)−EQimin⁡)]×αi=[ECimin⁡+EQKi×(EQ(i)−EQimin⁡)] ×[1+(DPK(i)−1)×EOKi],

where *α*_*i*_ = construction equipment cost modification factor during overtime because of extra or additional construction equipment, *α*_*i*_ = 1 + (DPK_(*i*)_ − 1) × EOK_*i*_; EOK_*i*_ = productivity decreased rate during overtime per unit time (e.g., hour), normally 20%.

### 3.4. Relationship between Construction Administration Quality and Administration Cost

A construction team consisting of sufficient crew members could improve construction quality and consume a reasonable cost [17], but the construction team hardly impacts on construction productivities. Therefore it is assumed that administration cost and administration quality are an approximate linear function:

(7) $$\begin{array}{c} {\mathrm{AC}}_{\left(i\right)}={\mathrm{AC}}_{i}^{\min}+{\text{AQK}}_{i}\times \left({\text{AQ}}_{\left(i\right)}-{\text{AQ}}_{i}^{\min}\right), \end{array}$$

where AQ_(*i*)_ = actual quality level of construction administration (*i*) in activity (*i*), AQ_(*i*)_ ∈ (AQ_*i*_^min⁡^, AQ_*i*_^max⁡^); AQ_*i*_^min⁡^ = minimum quality level of construction administration (*i*) in activity (*i*); AQ_*i*_^max⁡^ = maximum quality level of construction administration (*i*) in activity (*i*); AQK_*i*_ = (AC⁡_*i*_^max⁡^ − AC⁡_*i*_^min⁡^)/(AQ_*i*_^max⁡^ − AQ_*i*_^min⁡^); AC⁡_*i*_^min⁡^ = minimum cost of construction administration (*i*) in activity (*i*); AC⁡_*i*_^max⁡^ = maximum cost of construction administration (*i*) in activity (*i*); AC⁡_(*i*)_ = actual cost of construction administration (*i*) in activity, AC⁡_(*i*)_ ∈ (AC⁡_*i*_^min⁡^, AC⁡_*i*_^max⁡^).

Since work overtime might increase administration cost, the construction administration cost will be modified by factor *β*_*i*_:

(8) AC⁡(i)=[AC⁡imin⁡+AQKi×(AQi−AQimin⁡)]×βi=[AC⁡(i)=AC⁡imin⁡+AQKi×(AQ(i)−AQimin⁡)] ×[ACRKi+1−ACRKiDPK(i)],

where *β*_*i*_ = administration cost modification factor during work overtime because of extra or additional construction equipment, *β*_*i*_ = ACRK_*i*_ + (1 − ACRK_*i*_)/DPK_(*i*)_; ACRK_*i*_ = administration hourly cost rate factors in activity (*i*) when overtime working is applicable, usually 2.0.

### 3.5. Calculating Labor Cost and Activity Time

When labors are working together as a construction crew in a construction activity (*i*), their working duration is the time of the construction activity, which could be estimated by the construction quantities (QNT) and the actual productivity, overtime factors:

(9) $$\begin{array}{c} \text{DU}{\text{R}}_{\left(i\right)}=\frac{\text{QN}{\text{T}}_{\left(i\right)}}{\text{PR}{\text{D}}_{\left(i\right)}\times \text{DP}{\text{K}}_{\left(i\right)}}, \end{array}$$

where DUR_(*i*)_ = duration (time) of construction activity (*i*); QNT_(*i*)_ = quantities of construction activity (*i*); PRD_(*i*)_ = actual productivity in activity (*i*); DPK_(*i*)_ = overtime factors if accelerated construction speed is desired, DPK_(*i*)_ ∈ [1.0, 1.5] when overtime varies from 0 to 4 hours per day since standard working time is eight hours per day. DPK_(*i*)_ = 1.0 means no overtime work assigned.

Labor cost in construction activity (*i*) would be determined by standard daily cost and overtime work cost [18]. It is assumed that work overtime is paid in an hourly cost rate comparing with standard labor cost:

(10) LC(i)=LCDiS×DUR(i)+(DPK(i)−1) ×(LCRK(i)×LCDiS)×DUR(i)=LCDiS×DUR(i)×[1+(DPK(i)−1)×LCRK(i)]=(LCDiS×QNT(i)) ×([LPRDimax⁡−LQKi×(LQ(i)−LQimin⁡)]×[DEKimin⁡+DQKi×(EQ(i)−EQimin⁡)])−1 ×(LCRKi+1−LCRKiDPK(i)),

where LC_(*i*)_ = labor cost in construction activity (*i*); LCD_*i*_^*s*^ = standard labor cost per unit time (e.g., day) in construction activity (*i*); LCRK_*i*_ = labor cost rate factors in activity (*i*) when overtime work is applicable, usually 2.0.

## 4. Formulating PTCQTP Model

The purpose of formulation of a PTCQTP model is to optimize comprehensive construction time-cost-quality problem and to provide construction managers with a deliberate tool to balance critical construction resources in competitive construction industry. The project quality, time, and cost are quantified as follows.

### 4.1. Calculating Overall Quality

Since a construction project comprises various resources such as materials, machines, method, labors, and even management, the overall quality *Q* of a construction project is calculated by each activity's quality AQP_(*i*)_ and its quality weight WT_*i*_:

(11) $$\begin{array}{c} Q=\overset{n}{\underset{i=1}{\sum }}\left({\text{WT}}_{i}\times \text{AQ}{\text{P}}_{\left(i\right)}\right), \end{array}$$

where *Q* = the general quality of a construction project; *n* = number of activities in a construction project; WT_*i*_ = quality weight indicator of each construction activity (*i*), and ∑_*i*=1_^*n*^WT_*i*_ = 1.0; AQP_(*i*)_ = quality performance of construction activity (*i*) calculated by its labor quality, material quality, construction equipment quality, and administration quality, AQP_(*i*)_ = LWT_*i*_ × LQ_(*i*)_ + MWT_*i*_ × MQ_(*i*)_ + EWT_*i*_ × EQ_(*i*)_ + AWT_*i*_ × AQ_(*i*)_; LWT_*i*_, MWT_*i*_, EWT_*i*_, AWT_*i*_ = weight indicators of construction labor, material, equipment, and administration in activity (*i*) respectfully; LQ_(*i*)_, MQ_(*i*)_, EQ_(*i*)_, AQ_(*i*)_ = quality of construction labor, material, equipment, and administration in activity (*i*) respectfully.

### 4.2. Calculating Overall Cost

The overall cost of a construction project is added up with each construction activity's cost and its administration cost AC⁡_(*i*)_. Thereby the overall cost *C* is calculated as follows:

(12) $$\begin{array}{c} C=\overset{n}{\underset{i=1}{\sum }}\left({\text{LC}}_{\left(i\right)}+{\text{MC}}_{\left(i\right)}+{\text{EC}}_{\left(i\right)}+{\mathrm{AC}}_{\left(i\right)}\right), \end{array}$$

where *C* = the overall cost of a construction project; LC_(*i*)_, MC_(*i*)_, EC_(*i*)_, AC⁡_(*i*)_ = the cost of labor, materials, equipment, and administration in construction activity (*i*) respectfully; *n* = number of all construction activities.

### 4.3. Calculating Overall Time in Construction Project

The overall time *T* of a construction project can be easily calculated by nodes as activities in an acyclic digraph network:

(13) $$\begin{array}{c} T=\underset{i=1,n}{\max}\left(\text{ES}{\text{T}}_{\left(i\right)}+\text{Du}{\text{r}}_{\left(i\right)}\right), \end{array}$$

where *T* = overall time in construction project, EST_(*i*)_ = max⁡_*h*=1,*i*−1_(EST_(*h*)_ + Dur_(*h*)_), as well as the earliest starting time of activity (*i*) derived by its predecessors, and the first EST_(1)_ = 0.

## 5. Implementation of the PTCQTP Model

The PTCQTP model is to minimize the project overall time while conforming to the requirements of construction quality standards within a specified budget, which can be sated as follows:

(14) min⁡⁡ Z1=T, s.t.  C·X≤A;  Q·X≥B,

where *A* = the specified maximum cost; *B* = the minimum requirements of construction quality; X = [DPK_(1)_ ⋯ DPK_(*n*)_LQ_(1)_ ⋯ LQ_(*n*)_MQ_(1)_ ⋯ MQ_(*n*)_EQ_(1)_ ⋯ EQ_*n*_AQ_(1)_ ⋯ AQ_(*n*)_]_1×5*n*_^*T*^, a vector of all variables in the PTCQTP model.

In order to solve this comprehensive time-cost-quality tradeoff problem, a global optimization algorithm is necessary. The genetic algorithm is widely applied in optimization solution and pattern search is one of direct search methods, both methods can solve global optimization problems. Tests of different algorithms developed for this model reveal that the pattern search algorithm cannot solve this complex nonlinear programming problem by direct search which often falls into local optimization, but the genetic algorithm can find out a global optimization though solutions are not precise but acceptable.

The genetic algorithm has been widely applied in previous works of the literature; thereafter the development process of genetic algorithm for the PTCQTP model is not worthy of detailing here.

## 6. Example Illustration and Discussion

### 6.1. Example Illustration

**Figure 2:**
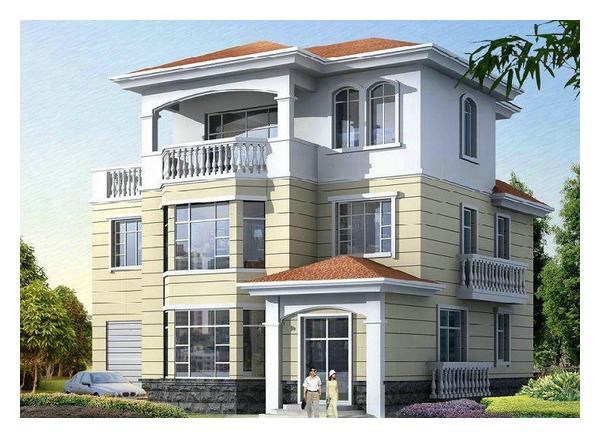
A three-story house as a study example.

Here is a typical brick concrete house with a concrete raft slab foundation and three stories as a study example to illustrate application of the PTCQTP model, shown in Figure 2. Construction lot is nearly 300 square meters (m^2^), and depth of the shallow foundation is one-half meter and earthwork volume needed to be moved is 240 m^3^. The first floor is 120 m^2^, the second floor is about 105 m^2^, and the third floor is 90 m^2^.

**Figure 3:**
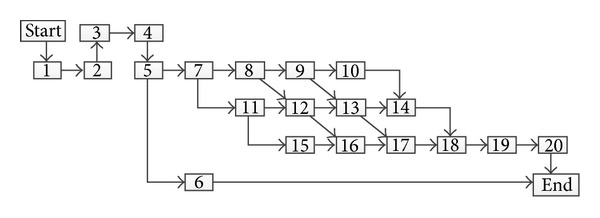
Construction procedure of the building example. 1: clear lot and excavate for foundation; 2: erect formwork for foundation concrete; 3: install foundation reinforcing; 4: pour footings; 5: build foundation block; 6: refill foundation earth; 7: erect formwork for 1st story; 8: install reinforcing for 1st story; 9: pour columns, beam, and slabs for 1st story; 10: build block in 1st story; 11: erect formwork for 2nd story; 12: install reinforcing for 2nd story; 13: pour columns, beam, and slabs for 2nd story; 14: build block in 2nd story; 15: erect formwork for 3rd story; 16: install reinforcing for 3rd story; 17: pour columns, beam, and slabs for 3rd story; 18: build block in 3rd story; 19: install heat insulation in the roof; 20: install waterproof layers for the roof.

The building consists of 20 construction activities, and the construction procedure is shown in an activity-on-node network in Figure 3. Each activity has a number of possible resource options and work options that can be used to construct the activity as shown in Table 1. Construction resources in each activity are labors, materials, equipment, and administration. Normal working shift is eight hours a day and the maximum working overtime is additional four hours a day. During working overtime, construction labors and administrators will be paid doubly (LCRK_*i*_ = 2.0 and ACRK_*i*_ = 2.0).

Since construction materials weigh heavily on the overall quality of a construction project, the quality weight indicators of construction materials are bigger than any other indicators. In this case the quality weight indicators of administration are the least important when comparing with other indicators.

After deliberating on all construction activities, 20 activities are grouped as 7 works: earth work, foundation work, 1st story work, 2nd story work, 3rd story work, and roof work. The quality weight indicators of group works (GWT_*j*_) are suggested first and then the quality weight indicators of all 20 construction activities (AWT_*i*_) are assigned from the quality weight indicators of their groups. All quality weight indicators applied in this building case are shown in Table 2.

### 6.2. Discussion

1. If *Q* = 0.8 or above and *C* = $350000 or below.

If the overall quality objective is 0.8 or above and the overall cost objective is $350000 or below, the optimal overall time is 64 days suggested by the PTCQTP model.

**Figure 4:**
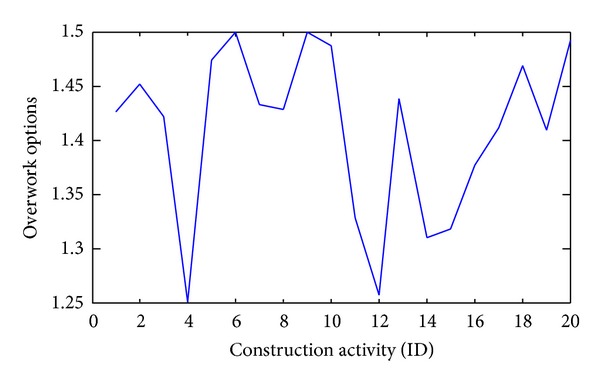
Optimized working hours a day in twenty activities.

All optimized construction arrangements and resource utilizations suggested by this model are available; for example, the optimal working hours of each activity are shown in Figure 4. The optimal working arrangement reveals that nearly all activities are carried out in overtime working hours since the construction time is limited. Although overtime working demands higher labor cost, overtime working shortens construction time and secures construction quality.

**Figure 5:**
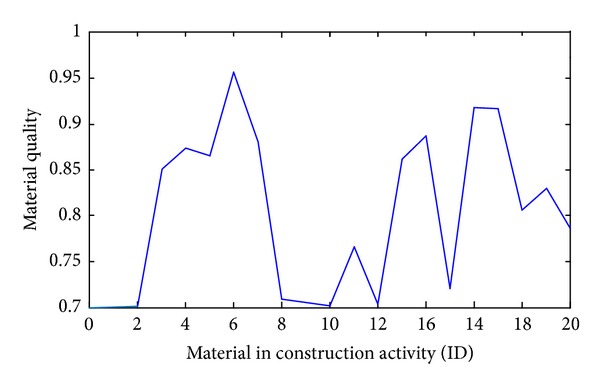
Optimized material quality performances in twenty activities.

The optimized material quality performances in 20 construction activities vary much, as shown in Figure 5. For example, materials in the activity (6) “-Refill foundation earth” are required to maintain the highest quality level ( = 0.95), while the activity (10) “-Build block in 1st story” and (12) “-Install reinforcing for 2nd story” only need to be the lowest quality level ( = 0.7). Obviously the lowest quality requirement would save cost.

(2) Optimized overall time-cost-quality.

**Figure 6:**
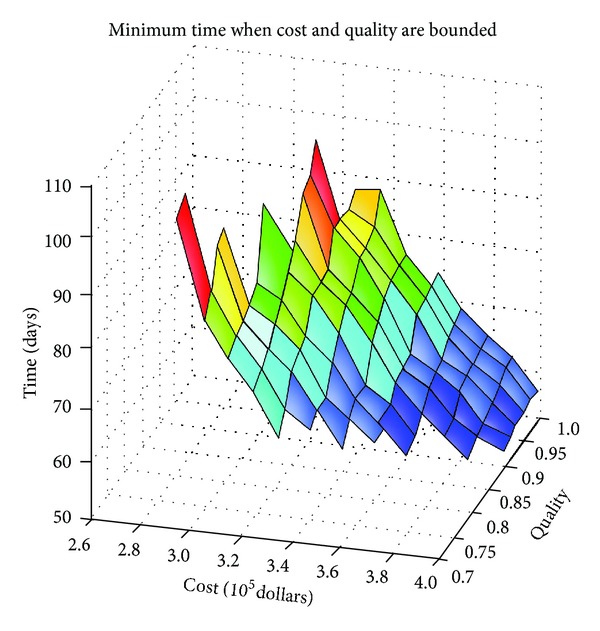
A visual three-dimensional tradeoff pattern among project time, cost, and quality.

A part of optimized results (shown in Table 3) are excerpted from numerous calculations when construction cost and quality are confined within potential solution boundaries. A visual tradeoff among construction time, cost, and quality is presented in Figure 6.

When a moderate quality performance, for example, 0.86, is set and the overall cost is $320,000, the overall time is about 90 days by the tradeoff model. The overall cost will climb to $390,000 if the overall time is shortened to 54 days and the quality objective is unchanged.

**Figure 7:**
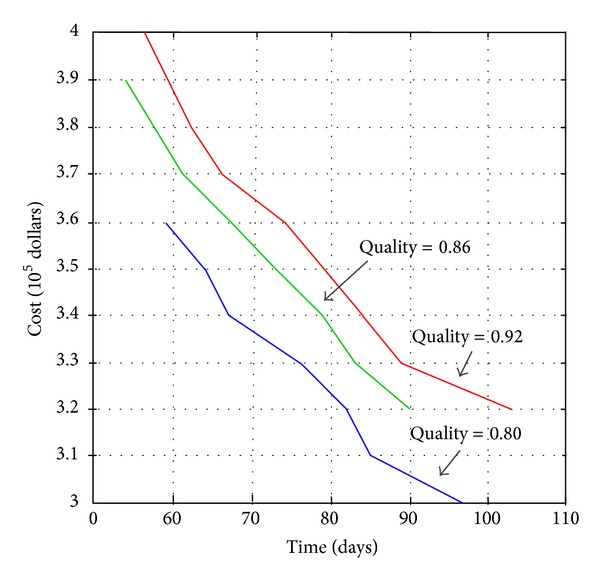
Tradeoff relationship between construction cost and time when the quality sets 0.80, 0.86, and 0.92.

Tradeoff curves between the overall cost and time under different quality objectives are shown in Figure 7, which proves that traditional linear assumptions between project cost and time are reasonable somehow.

## 7. Conclusion

Project time-cost-quality tradeoff is a complex nonlinear optimization problem that is easily understood in concepts but seldom solved in practices. All construction managers and engineers are eagerly searching for feasible methods to solve it. Base on breakdown structures of construction project and discussion of time-cost-quality relationship of construction activities, an advanced PTCQTP Model is developed in this paper to search for optimal resource utilization arrangement that results in minimizing project time within a certain budget while maximizing construction quality. Construction resources comprise labors, materials, equipment, and administration. Each construction resource varies in quality, cost, and productivity which eventually determine construction time. A genetic algorithm is integrated into the PTCQTP model to solve this complex nonlinear program problem. Arrangement of resource utilizations is visualized in graphics that helps construction managers make tradeoff decisions better.

An application example of a three-story house construction is introduced to illustrate the implementation of the PTCQTP model and demonstrate its advantages in optimizing tradeoff of construction time, cost, and quality. The example provides useful three-dimensional and two-dimensional visual relationships among project time, cost, and quality and resource utilization planning which enable construction managers and engineers to make a winning decision in fiercely construction competition. The computational time-cost-quality curves in visual three-dimensional graphics from the case study prove traditional cost-time assumptions reasonable and also prove this time-cost-quality tradeoff model sophisticated.

Future studies are to assume more sophisticated relationships among the cost, time, and quality of different project resources, test more project cases needed, and find out other more efficient optimal algorithms.

## Figures and Tables

**Table 1:** Construction activities with resource and work options.

Activity group	Activity ID	Quantity (QNT_*i*_)	Productivity (LPRD_min_–LPRD_max_)	Overtime factor (DPK_*i*_)	Labor			Material		Equipment			Administration		
					Cost rate (LCR_*i*_) ($/d)	Labor cost (LC_*i*_)	Labor quality (LQ_min_–LQ_max_)	Cost (MC_min_–MC_max_) ($)	Quality (MQ_min_–MQ_max_)	Cost (EC_min_–EC_max_) ($)	Quality (EQ_min_–EQ_max_)	Productivity factor (DEK_min_–DEK_max_)	Cost rate (ACR_min_–ACR_max_) ($/d)	Quality (AQ_min_–AQ_max_)	Cost (AC⁡_*i*_) ($)
Earth work	1	240 m^3^	100–150 m^3^/d	1–1.5	700	—	0.8–1.0	3200–4500	0.7–1.0	600–650	0.8–1.0	0.8–1.2	90–150	0.7–1.0	—

Foundation	2	110 m^2^	10–12 m^2^/d	1–1.5	300	—	0.8–1.0	800–1100	0.7–1.0	110–120	0.8–1.0	0.98–1.02	90–150	0.7–1.0	—
	3	1.6 t	0.6–0.8 t/d	1–1.5	600	—	0.7–1.0	5600–7200	0.7–1.0	128–144	0.8–1.0	0.9–1.1	90–150	0.7–1.0	—
	4	52 m^3^	15–20 m^3^/d	1–1.5	750	—	0.9–1.0	18200–23400	0.7–1.0	150–180	0.9–1.0	0.95–1.05	90–150	0.7–1.0	—
	5	75 m^3^	8–12 m^3^/d	1–1.5	1700	—	0.8–1.0	16000–20000	0.7–1.0	1500–1800	0.8–1.0	0.98–1.02	90–150	0.7–1.0	—
	6	60 m^3^	20–25 m^3^/d	1–1.5	430	—	0.8–1.0	6400–8600	0.7–1.0	120–150	0.8–1.0	0.9–1.1	90–150	0.7–1.0	—

1st story	7	120 m^2^	8–11 m^2^/d	1–1.5	450	—	0.7–1.0	1200–1700	0.7–1.0	210–230	0.9–1.0	0.98–1.02	90–150	0.7–1.0	—
	8	3.2 t	0.8–1.2 t/d	1–1.5	800	—	0.7–1.0	11200–14400	0.7–1.0	256–288	0.8–1.0	0.9–1.1	90–150	0.7–1.0	—
	9	48 m^3^	7–10 m^3^/d	1–1.5	1000	—	0.7–1.0	16800–21600	0.7–1.0	200–240	0.9–1.0	0.95–1.05	90–150	0.7–1.0	—
	10	62 m^3^	4–6 m^3^/d	1–1.5	750	—	0.8–1.0	13200–16600	0.7–1.0	1240–1488	0.8–1.0	0.98–1.02	90–150	0.7–1.0	—

2nd story	11	105 m^2^	8–11 m^2^/d	1–1.5	460	—	0.7–1.0	1100–1600	0.7–1.0	180–200	0.8–1.0	0.98–1.02	90–150	0.7–1.0	—
	12	2.8 t	0.8–1.2 m^2^/d	1–1.5	800	—	0.7–1.0	9800–12600	0.7–1.0	224–252	0.8–1.0	0.9–1.1	90–150	0.7–1.0	—
	13	43 m^3^	7–10 m^3^/d	1–1.5	1000	—	0.7–1.0	15050–19350	0.7–1.0	180–210	0.9–1.0	0.95–1.05	90–150	0.7–1.0	—
	14	55 m^3^	4–6 m^3^/d	1–1.5	750	—	0.8–1.0	11700–14700	0.7–1.0	1100–1320	0.8–1.0	0.95–1.05	90–150	0.7–1.0	—

3rd story	15	95 m^2^	7–10 m^2^/d	1–1.5	470	—	0.7–1.0	1100–1600	0.7–1.0	180–210	0.8–1.0	0.95–1.05	90–150	0.7–1.0	—
	16	2.2 t	0.8–1.2 t/d	1–1.5	800	—	0.7–1.0	7700–9900	0.7–1.0	176–198	0.8–1.0	0.9–1.1	90–150	0.7–1.0	—
	17	38 m^3^	7–10 m^3^/d	1–1.5	1000	—	0.7–1.0	13300–17100	0.7–1.0	175–190	0.9–1.0	0.95–1.05	90–150	0.7–1.0	—
	18	50 m^3^	4–6 m^3^/d	1–1.5	750	—	0.8–1.0	10600–13300	0.7–1.0	1000–1200	0.8–1.0	0.9–1.1	90–150	0.7–1.0	—

Roof	19	135 m^2^	18–24 m^2^/d	1–1.5	300	—	0.7–1.0	3200–3800	0.7–1.0	40–50	0.8–1.0	0.98–1.02	90–150	0.7–1.0	—
	20	160 m^2^	40–50 m^2^/d	1–1.5	750	—	0.7–1.0	7000–8500	0.7–1.0	100–120	0.8–1.0	0.98–1.02	90–150	0.7–1.0	—

**Table 2:** The quality weight indicators.

Group works	Quality weight indicators of group works (GWT_*j*_)	Activity (*i*) (ID)	Quality weights indicators of activity (*i*) (AWT_*i*_)	Quality weight indicators of labors (*i*) (LWT_(*i*)_)	Quality weight indicators of materials (*i*) (MWT_(*i*)_)	Quality weight indicators of equipment (*i*) (EWT_(*i*)_)	Quality weight indicators of administration (*i*) (AWT_(*i*)_)
Earth work	0.05	1	0.05	0.1	0.7	0.15	0.05

Foundation work	0.25	2	0.05	0.1	0.7	0.15	0.05
		3	0.06	0.1	0.7	0.15	0.05
		4	0.06	0.1	0.7	0.15	0.05
		5	0.06	0.1	0.7	0.15	0.05
		6	0.02	0.1	0.7	0.15	0.05

1st story work	0.2	7	0.05	0.1	0.7	0.15	0.05
		8	0.05	0.1	0.7	0.15	0.05
		9	0.05	0.1	0.7	0.15	0.05
		10	0.05	0.1	0.7	0.15	0.05

2nd story work	0.2	11	0.05	0.1	0.7	0.15	0.05
		12	0.05	0.1	0.7	0.15	0.05
		13	0.05	0.1	0.7	0.15	0.05
		14	0.05	0.1	0.7	0.15	0.05

3rd story work	0.15	15	0.03	0.1	0.7	0.15	0.05
		16	0.04	0.1	0.7	0.15	0.05
		17	0.04	0.1	0.7	0.15	0.05
		18	0.04	0.1	0.7	0.15	0.05

Roof work	0.15	19	0.07	0.1	0.7	0.15	0.05
		20	0.08	0.1	0.7	0.15	0.05

*∑*	1.0	—	1.0	—	—	—	—

**Table 3:** The minimum time of a building project while project cost and general quality are bounded.

Quality	Time (days)												
	Cost (×10^5^ dollars)												
	2.8	2.9	3.0	3.1	3.2	3.3	3.4	3.5	3.6	3.7	3.8	3.9	4.0
0.74	109	101	84	78	72	65	—	—	—	—	—	—	—
0.76	—	105	88	80	75	68	66	—	—	—	—	—	—
0.78	—	—	94	81	77	73	67	60	—	—	—	—	—
0.80	—	—	97	83	80	76	67	64	61	—	—	—	—
0.82	—	—	—	85	82	76	72	66	62	57	—	—	—
0.84	—	—	—	97	85	82	76	67	64	59	—	—	—
0.86	—	—	—	—	90	83	79	73	67	61	58	54	—
0.88	—	—	—	—	97	84	79	75	67	64	60	57	54
0.90	—	—	—	—	98	89	84	77	68	65	62	57	56
0.92	—	—	—	—	103	89	84	79	74	66	63	59	56
0.94	—	—	—	—	—	90	86	80	75	68	64	61	56
0.96	—	—	—	—	—	91	91	80	75	69	65	62	56
0.98	—	—	—	—	—	—	—	—	76	69	65	62	57
1.00	—	—	—	—	—	—	—	—	—	—	—	—	—

Note: — means not available.
